# New Type of Halogen Bond: Multivalent Halogen Interacting with π- and σ-Electrons

**DOI:** 10.3390/molecules22122150

**Published:** 2017-12-05

**Authors:** Sławomir J. Grabowski

**Affiliations:** 1 Faculty of Chemistry, University of the Basque Country and Donostia International Physics Center (DIPC), P.K. 1072, 20080 Donostia, Spain; s.grabowski@ikerbasque.org; Tel.: +34-943-01-5477; 2 IKERBASQUE, Basque Foundation for Science, 48011 Bilbao, Spain

**Keywords:** electron charge shifts, halogen bond, octet rule, hydrogen bond, σ-hole bond

## Abstract

MP2/aug-cc-pVTZ calculations were performed for complexes of BrF_3_ and BrF_5_ acting as Lewis acids through the bromine centre, with species playing a role of Lewis base: dihydrogen, acetylene, ethylene, and benzene. The molecular hydrogen donates electrons by its σ-bond, while in remaining moieties—in complexes of hydrocarbons; such an electron transfer follows from π-electrons. The complexes are linked by a kind of the halogen bond that is analyzed for the first time in this study, i.e., it is the link between the multivalent halogen and π or σ-electrons. The nature of such a halogen bond is discussed, as well as various dependencies and correlations are presented. Different approaches are applied here, the Quantum Theory of Atoms in Molecules, Natural Bond Orbital method, the decomposition of the energy of interaction, the analysis of electrostatic potentials, etc.

## 1. Introduction

The halogen bond is one of the most important interactions that play a crucial role in numerous chemical and biological processes; it is analyzed in experimental and theoretical studies [1,2,3,4]. For example, its role in crystal engineering was described, and it was found that it is often competitive with the hydrogen bond [5]. The nature of halogen bond was discussed and it was compared with other Lewis acid—Lewis base interactions, especially with the hydrogen bond [6,7]. It is important that the halogen atom, X, often possesses dual character, and it may act as the Lewis acid and as the Lewis base centre simultaneously [8]. Particularly, such dual character is observed for C–X bonds; if the halogen’s acidic properties are revealed, then thus the C–X...B halogen bond is often observed; C is the carbon atom, while B is the Lewis base centre that is rich of electron charge. In general, the term A–X...B may be attributed to the halogen bond (A is the part of the Lewis acid moiety). In a case of halogen centre acting as the Lewis base there are the interactions which may be classified as halide bonds [9].

The dual character of halogen atoms may be explained in terms of the σ-hole concept that is applied for the halogen bond [3,4], as well as for other interactions where the centers of groups 14–18 act as Lewis acids in spite that numerous of them are classified as electronegative ones [6,10,11]. The latter atoms often possess areas of the positive electrostatic potential (EP) since the electron density for A–Z bonds (Z is attributed to tetrel, pnicogen, chalcogen, halogen or aerogen centre) is moved from the Z-centre to the A–Z bond and to other parts of the Lewis acid species. That is why the positive EP may be observed in the extension of the A–Z bond, and the A–Z...B interactions classified as the σ-hole bonds are usually linear or nearly so.

In numerous interactions, π-electron systems are those that play a role of Lewis bases. Especially, such bases were analyzed for hydrogen bonded systems and it was found that the C–H...π hydrogen bonds are often observed in crystal structures influencing strongly an arrangement of molecules [12]. In general, if the Lewis base is considered, thus the following types of hydrogen bonds may be specified; A–H...B with the single Lewis base centre, B; A–H...π, where the multicenter Lewis base is observed that is the π-electron system such as: acetylene, ethylene, benzene, and their numerous derivatives; finally, the A–H...σ hydrogen bonds may be specified where σ-electrons play a role of the Lewis base [13]; it seems that such interactions exist only if molecular hydrogen acts as a base [14,15]. It was pointed out recently, that those types of Lewis bases are observed not only for the hydrogen bonded systems, but also for other interactions, especially for the σ-hole bonds, such as the pnicogen, chalcogen, halogen bonds, etc. [9]. The same concerns the triel bonds, the sub-class of the π-hole bonds, since the triel...π/σ-electron interactions were analyzed theoretically, and even these interactions are observed in crystal structures [16,17].

The aim of this study is an analysis of a special kind of halogen bond, where on one hand, π-electron or σ-electron systems donate electrons, and on the other hand, multivalent halogen centers play a role of Lewis acids. There are only few studies on halogen bonds with the π-electron species as the Lewis bases; also studies on halogen bonds with multivalent halogen centers are very rare. However, it seems the multivalent halogen—π/σ-electrons interactions were not analyzed yet.

One can mention following examples of studies on the A–X...π halogen bonds; theoretical calculations on the X...π systems where acetylene plays a role of the Lewis base [18]; ab initio calculations and the QTAIM approach were applied for the C_2_H_4_...ClF and H_2_...ClF systems [19]; crystal structures were analyzed where interactions of bromine, Br_2_, with π-electrons of benzene and toluene occur [20], the hydrogen and halogen bonds were compared, i.e., H...π, F...π and Cl...π were considered [21]; different halogen bonds were analyzed in terms of various approaches, among these interactions systems with π-electron Lewis bases were considered [22]; the directionality of halogen–borazine interactions was analyzed and such interactions were classified as the A–X...π halogen bonds [23]; the P...π pnicogen bonds were compared with the Br...π halogen bonds [24] or very recently different types of the σ-hole bonds were analyzed, among them, halogen bonds with acetylene playing a role of the Lewis base [25].

The similar situation occurs for interactions of multivalent halogens, only several studies were carried out; [Ph_2_IX]_2_ dimers were early analyzed theoretically (X = Cl, Br, I) where the trivalent iodine centre was considered [26]; the intermolecular hypervalent I(III)...O interactions were described by M. Ochiai [27]; theoretical calculations on the halogen multivalent centers acting as the Lewis acids [28] were supported by searches that were performed through the Cambridge Structural Database [29]; B3LYP/aug-cc-pVTZ calculations were carried out for the deprotonated 2-iodoxybenzoic acid and its analogs, and it was found that these systems are stabilized partly due to interactions where the multivalent iodine plays a role of the Lewis acid center [30]; the selenium, arsenic, and phosphorus hypervalent centers in oxyanions were also considered in this study as those that can interact with the electron rich sites [30]. In general, it was noted that σ-holes for Group IV-VII atoms in some hypervalent configurations are observed [31]. One can also mention other more recent studies where the complexes of BrF_3_ and BrF_5_ species were considered [32]; where complexes of FXO*_n_* (X = Cl, Br; *n* = 0–3)-CH_3_CN were analyzed [33]; or the study where a special attention was paid on the electrostatic potentials of the hypervalent halogen centers and numerous examples taken from the Cambridge Structural Database (CSD) were presented [34]. The analysis of the Lewis acid properties of hypervalent halogen fluorides [35] was carried out recently in terms of the hole-lump concept [36]. There is also a recent interesting study on the charge assisted halogen bond, where the bromonium and iodonium cations possess the σ-holes at the multivalent halogens (Br or I), which may act as the Lewis acid centers [37]; the evidence of bifurcated halogen bonds for such hypervalent halogens was also found in few crystal structures [37]. The latter study that considers mainly arrangements in numerous crystal structures was supported by M06-2X/6-311g(d) calculations.

The analysis of the halogen (multivalent)...π/σ-electrons interactions is performed here and it is supported by various approaches; the Quantum Theory of Atoms in Molecules (QTAIM) [38], Natural Bond Orbital (NBO) approach [39], the decomposition of the energy of interaction [40,41], as well as the analysis of the electrostatic potential (EP) distribution [42].

## 2. Results and Discussion

### 2.1. Energetic and Geometric Parameters

**Figure 1:**
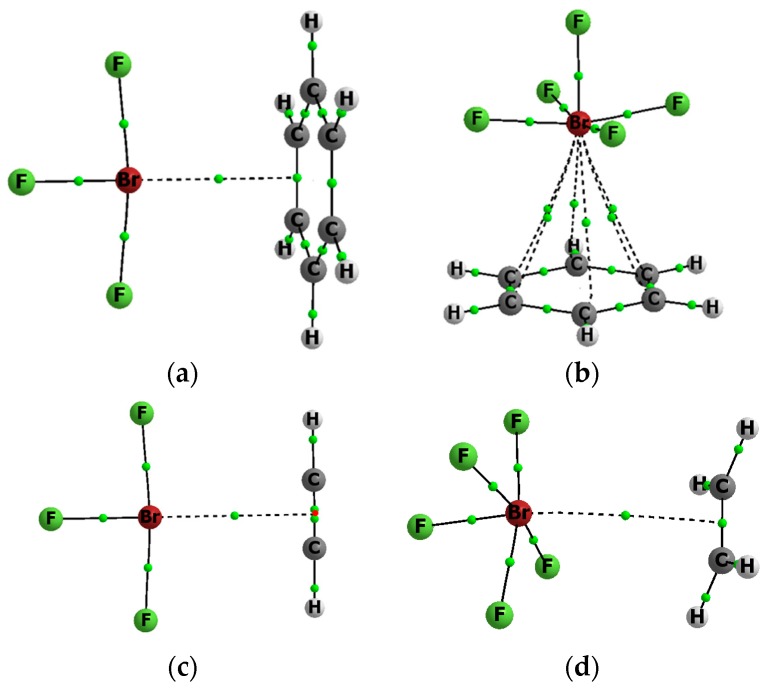
The molecular graphs of the following complexes; (**a**) BrF_3_-C_6_H_6_; (**b**) BrF_5_–C_6_H_6_; (**c**) BrF_3_–C_2_H_2_; and, (**d**) BrF_5_–C_2_H_4_; big circles—attractors, small green circles—BCPs, for the BrF_3_–C_2_H_2_ complex (**c**), the NNA is located (small red circle) between two BCPs.

Figure 1 presents examples of molecular graphs of selected complexes that are analyzed in this study. Both complexes with benzene are shown, as well as the BrF_3_…C_2_H_2_ and BrF_5_…C_2_H_4_ complexes. For the BrF_5_…C_2_H_4_ complex, the F-Br…BCP arrangement is not linear. It is connected with the electrostatic potential distribution for the BrF_5_ species, four positive maxima of EP at the Br-centre are observed here that are related by the four-fold axis of symmetry passing through the axial F-Br bond [32]. For the BrF_3_ species the EP maximum is located in the elongation of the axial F-Br bond [32], or nearly so [34]; that is why the linear F-Br…NNA (NNA: non-nuclear attractor) arrangement is observed here (Figure 1).

The binding and interaction energies for analyzed complexes are presented in Table 1. One can see very weak A–X...σ halogen bonds for complexes with dihydrogen, -E_int_ and -E_bin_ do not exceed 1 kcal/mol. The A–X...π halogen bonds are much stronger interactions than their counterparts with dihydrogen acting as the Lewis base. Particularly relatively strong interactions for complexes with benzene are observed where -E_int_ amounts ~9 kcal/mol, more than for hydrogen bond in water dimer, where such a value is equal to ~4–5 kcal/mol [43]. For the BrF_3_ complexes stronger interactions are observed than for their BrF_5_ analogues, except of the benzene complexes where in a case of the BrF_5_ complex slightly stronger halogen bond occurs.

The negligible values of deformation energy, E_def_, are observed; they do not exceed 0.5 kcal/mol. It means that the complexation rather does not influence on geometries of monomers which participate in halogen bonds analyzed here; especially in a case of the weakest interactions with dihydrogen. The basis set superposition error (BSSE) correction seems to be important in a case of systems that are analyzed in this study; particularly for the strongest interactions with benzene where it amounts 3.2–3.5 kcal/mol; in spite of the fact that rather large and saturated aug-cc-pVTZ basis set is applied. The greater BSSE corrections are observed for stronger interactions (Table 1); it is worth mentioning that there is the linear correlation between the interaction energy and BSSE (R = 0.97).

Table 1 presents also E_NBO_ energies that correspond to the π_CC_ → σ_BrF_* and σ_H2_ → σ_BrF_* overlaps for hydrocarbons and dihydrogen complexes, respectively (see the section on computational details). Such overlaps are observed for all BrF bonds thus the energies presented in the table are sums of the orbital-orbital energies for the complex considered. The greater orbital-orbital energies are observed for the axial BrF bonds than for their equatorial counterparts. It is worth mentioning that the axial BrF bonds for the BrF_3_ and BrF_5_ molecules that are characterized by the C_2v_ and C_4v_ symmetry are located on the 2-fold axis and on the 4-fold axis, respectively. The greater E_NBO_ energies are observed for the BrF_3_ complexes than for their BrF_5_ analogues. 

It is worth mentioning that meaningless, if any, elongations of the axial Br-F bonds as a result of complexation are observed while such elongations for the equatorial Br-F bonds are usually more important (Table 1SM with Br-F bond lengths is included in Supporting Material—SM). The above lengths’ changes, which result from the electron charge shifts do not correlate with the total interaction energy, while the second order polynomial correlation is observed for the relationship between percentage elongation of the equatorial Br-F bond and the NBO energy that is related to the π_CC_ → σ_BrF_* or σ_H2_ → σ_BrF_^*^ overlap (R^2^ = 0.9678, see Figure 1SM in SM). It means that interactions that are related to the electron charge density shifts are mainly responsible for the change of geometries of interacting species. In general the latter conclusion is in line with recent findings concerning the σ-hole bonds and the π-hole bonds [9].

The Lewis acid–Lewis base distances are also collected in Table 1; the values in the 2.84–3.22 Å range are observed, there is the shortest distance for the BrF_3_–C_6_H_6_ complex. These are distances between the Br-centre and the mid-point of the CC or HH bond for the BrF_3/5_…C_2_H_2/4_ and BrF_3/5_…H_2_ complexes, respectively. In a case of the BrF_3_…C_6_H_6_ complex it is the distance between the Br-centre and the mid-point of the nearest CC bond of benzene; in a case of the BrF_5_…C_6_H_6_ complex, it is the distance between the Br-centre and the centre of the benzene molecule (Figure 1).

### 2.2. Nature of Interactions–Decomposition of Interaction Energy

Table 2 presents the terms of the energy of interaction resulting from the Ziegler and Rauk decomposition scheme (see section on computational details) [41]. One can see that for the BrF_3_ complexes, electrostatic and orbital interactions are the most important attractive terms; they are equal one to each other or nearly so since the ratio between them, ΔE_elstat_/ΔE_orb_ is equal to 0.9–1.0. The dispersive interaction is less important for all of the complexes, both for BrF_3_ and for BrF_5_ ones. For the BrF_5_ complexes however, the electrostatic term is slightly more important since the ΔE_elstat_/ΔE_orb_ ratio is equal here 1.3; the BrF_5_–C_2_H_2_ complex is an exception since the orbital energy is more important here than electrostatic one.

**Figure 2:**
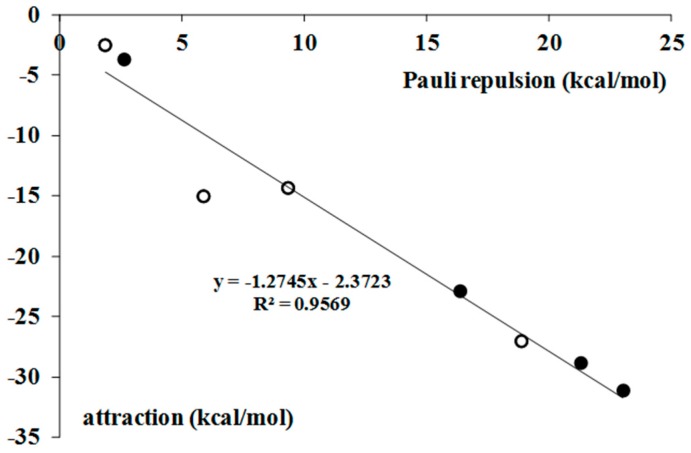
The linear correlation between the repulsion interaction energy and the sum of attractive terms (both in kcal/mol); black circles—the BrF_3_ complexes, white circles—the BrF_5_ complexes.

It was shown recently, for the σ-hole bonds, as well as particularly for the hydrogen bonds, that the formation of intermolecular link is accompanied by numerous effects that may be treated as the response of the complex for the Pauli repulsion [9]. Various correlations were found between the repulsion interaction energy and different terms of the attractive interaction. The same is observed here. Figure 2 presents the correlation between the Pauli repulsion interaction energy and the sum of the attractive terms (electrostatic, orbital and dispersion).

It seems to be a surprising result that the orbital interaction is often comparable here with the electrostatic interaction; however, it was found in earlier studies, especially those concerning hydrogen bonds, that in a case of π-electrons playing a role of the Lewis base in complexes, the interaction energy terms related to the electron charge shifts are very important [44,45].

### 2.3. QTAIM Parameters

Table 3 presents characteristics of the bond critical point (BCP) for the bond path linking the Lewis acid and Lewis base units in the complex considered. This is a link between the bromine centre and the critical point of the CC bond or of the HH bond. Only in a case of the BrF_5_–C_6_H_6_ complex (see Figure 1), the bromine attractor is connected by six approximately equivalent bond paths with carbon attractors of benzene. For all of the other complexes, the Br-attractor is connected with the non-nuclear attractor (NNA), which is located between two BCPs of CC bond (like for the BrF_3_–C_2_H_2_ complex in Figure 1) or with the BCP of the CC/HH bond (the BrF_3_–C_6_H_6_ and BrF_5_–C_2_H_4_ complexes in Figure 1 are examples). It was found in earlier studies that there are bond paths between typical atom attractors and NNAs or BCPs of π/σ-electron systems. Such cases were analyzed for the A–H...π and A–H...σ hydrogen bonds and latter for other complexes of π-electron or σ-electron species, which are linked by the σ-hole or π-hole bonds [15,46,47].

It was found in numerous studies that characteristics of BCP that correspond to the intermolecular link may be often treated as measures of the strength of interaction [45,48]. Especially it is in force for homogeneous samples of complexes; numerous relationships between the characteristics of the H...B BCP and the strength of interaction were found for the A–H...B hydrogen bonded systems. For the sample of complexes that are analyzed here, there is a well exponential correlation (R^2^ = 0.97) between the electron density of the above-mentioned BCP, *ρ*_BCP_, and the interaction energy (corrected for BSSE), E_int_, but if the complexes with benzene are excluded from this relationship. This is because the distinct links between monomers in benzene complexes exist if they are compared with the other systems analyzed here.

Table 3 shows that the greatest *ρ*_BCP_ value that is observed for the BrF_3_–C_2_H_4_ complex, this one characterized by the highest -E_int_ value (if benzene complexes are excluded), the lowest *ρ*_BCP_ values are observed for complexes of dihydrogen. The laplacian of the electron density at BCP, ∇^2^*ρ*_BCP_, is positive for all of the complexes analyzed that suggest these are not covalent in nature interactions; similarly, H_BCP_ values are positive and close to zero. Only for the BrF_3_–C_2_H_4_ complex H_BCP_ is negative but also close to zero, it is equal to −0.001 au. It seems that these results are not in agreement with those of the decomposition of the energy of interaction. The latter results show the comparable contributions of electrostatic and orbital interactions for complexes analyzed here. The orbital interaction energy term is often attributed to the covalent character of interaction. However, it is worth mentioning that the interaction energy terms that are attributed to covalency are often important for those complexes where the π-electron systems play a role of the Lewis base [44,45]. It was mentioned in the previous section that it was found for the hydrogen bonded systems.

### 2.4. Electron Charge Density Shifts

The complexation is always connected with the electron charge shift from the acidic unit to the basic one in a case of the Lewis acid–Lewis base interactions [9]. This transfer is usually greater for stronger interactions. For the complexes analyzed here, the greater transfer is observed for the BrF_3_ complexes than for the BrF_5_ counterparts (Table 4); approximately, it is in line with the binding and interaction energies since stronger interactions are observed for the BrF_3_ species. The latter conclusion is in force for transfers that are calculated from the NBO charges [39], as well as from the Hirshfeld charges [49]. If one considers the Lewis base units, thus there is the following order of the increase of the electron charge transfer: H_2_ < C_2_H_2_ < C_2_H_4_ ≈ C_6_H_6_; and again, the latter order works for the Hirshfeld and NBO charges.

Table 4 presents also the NBO atomic charges [39] of bromine centre. One can see that in a case of the BrF_3_ systems, the complexation leads to slight changes of the charge of bromine, sometimes the increase of the positive charge of this centre is observed (for complexes of dihydrogen and acetylene), sometimes the decrease of this charge (complexes of ethylene and benzene). For all of the BrF_5_ complexes, the halogen bond formation leads to the increase of the positive charge of the bromine centre. The latter is in line with the electron charge changes for the hydrogen bonded systems where the complexation leads to the increase of the positive charge of the Lewis acid central atom—the hydrogen [39,45].

The formation of the A–H...B hydrogen bond also results in the increase of the polarization of the A–H bond (see the section on computational details for the definition of the bond polarization). It means that the percentage of the electron density at the A-centre increases. For the species analyzed here, the meaningful changes of Br-F bond polarizations are observed for the equatorial bonds, while for the axial ones, they are negligible. The percentage increase as a result of complexation of the mean Br-F equatorial bond polarization for the complexes considered is given in Table 4. It concerns the increase of the electron density at equatorial fluorine in the complex in relation to such density in the Lewis acid unit, BrF_3_ or BrF_5_, which is not involved in any interaction. Table 4 does not show such the polarization increase for the BrF_5_–C_6_H_6_ complex where NBO approach shows the fixed Lewis acid structure with the single, axial Br-F bond orbital.

**Figure 3:**
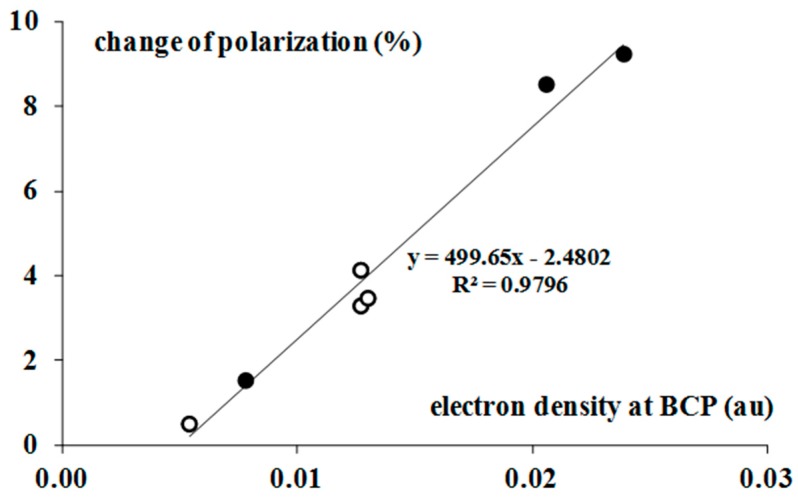
The linear correlation between the electron density at the BCP (in au) of the Lewis acid—Lewis base bond path, and the percentage change of the mean polarization of the Br-F equatorial bond as an effect of complexation; black circles correspond to the BrF_3_ complexes while white circles to the BrF_5_ complexes.

**Figure 4:**
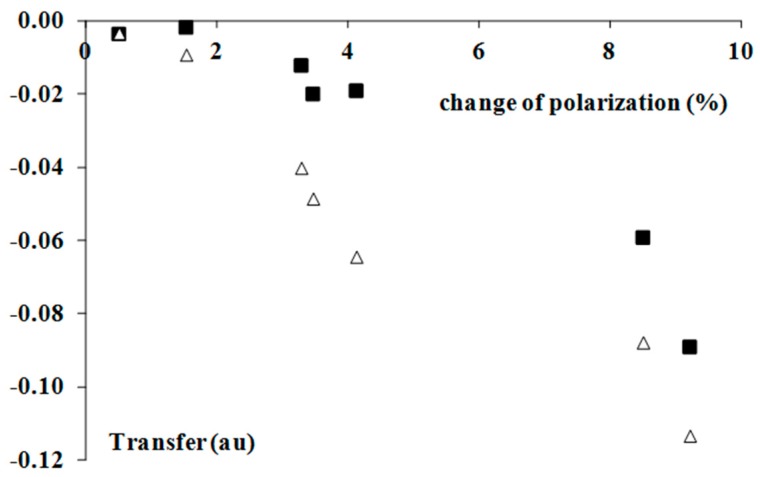
The dependencies between the percentage change of the mean polarization of the Br-F equatorial bond as an effect of complexation and the electron charge transfer from the Lewis base to the Lewis acid unit (in au); white triangles correspond to the Hirshfeld charges, while black squares to the NBO population.

The increase of the polarization of the Br-F equatorial bonds is in line with the other findings concerning the hydrogen bonds, and in general, the σ-hole bonds [9]. The additional interactions lead to electron density shifts, which try to protect the former octet (or doublet) structure of the Lewis acid centre that is not involved in interactions [9]. Hence, the outflow of the electron density from the Lewis acid centre is observed. Figure 3 shows the linear correlation between the *ρ*_BCP_ value, which expresses the strength of interaction and the above-mentioned percentage increase of the equatorial Br-F bond polarization. The stronger interactions lead to greater polarizations that try to protect the former octet Lewis structures. Figure 4 shows the tendency of the greater Br-F bonds polarization, which accompanies the greater electron charge transfer; such tendencies for both types of population analyses (NBO and Hirshfeld charges) are presented in this figure.

## 3. Computational Details

The calculations were performed with the Gaussian09 set of codes [50]; they were carried out using the second-order Møller-Plesset perturbation theory method (MP2) [51], and the Dunning style basis set, aug-cc-pVTZ [52]. Frequency calculations that were performed at the same computational level for the above-mentioned complexes and their monomers confirmed that the obtained structures correspond to energetic minima. The atoms’ coordinates for optimized complexes that are analyzed here are collected in Supporting Material. The binding energy, E_bin_, was calculated as the difference between the energy of the complex and the sum of energies of monomers optimized separately, while the interaction energy, E_int_, is a difference between the energy of the complex and the sum of energies of monomers which geometries come from the optimized complex [53]. Both those energies are negative, while the binding and interaction energies difference—the deformation energy, E_def_, is positive and it is connected with the change of geometries of monomers resulting from the complexation [54]. The Counterpoise (CP) correction was applied to assess BSSE [55], thus the E_bin_ and E_int_ values corrected for BSSE are considered in this study.

The Quantum Theory of ‘Atoms in Molecules’ (QTAIM) was also applied to characterize critical points (BCPs) in terms of the electron density (ρ_BCP_), its Laplacian (∇^2^ρ_BCP_), and the total electron energy density at BCP (H_BCP_); the latter energy is a sum of the potential electron energy density (V_BCP_) and the kinetic electron energy density (G_BCP_) [38]. The AIMAll program was used to perform the QTAIM calculations [56].

The Natural Bond Orbital (NBO) method [39] was applied to analyze atomic charges, as well as orbital-orbital interactions. The n_B_ → σ_AH_* overlap is considered as an interaction being characteristic for the A–H…B hydrogen bond; n_B_ designates here the lone electron pair of the B proton acceptor (the Lewis base); and, σ_AH_^*^ is an antibonding orbital of the proton donating bond (the Lewis acid) [39]. In a case of the A–H…π and A–H…σ hydrogen bonds, the π_B_ → σ_AH_* and σ_H2_ → σ_AH_* overlaps, respectively, are the most important orbital-orbital interactions [15]. The similar situation occurs for halogen bonds analyzed here where π_CC_ → σ_BrF_* and σ_H2_ → σ_BrF_* overlaps are the most important interactions. For example, the π_CC_ → σ_BrF_^*^ interaction is calculated as the second-order perturbation theory energy (Equation (1)):

ΔE (π_CC_ → σ_BrF_*) = −2 〈π_CC_|*F*|σ_BrF_*〉^2^/(ε (σ_BrF_*) − ε (π_CC_)), (1)

〈π_CC_|*F*|σ_BrF_*〉 designates the Fock matrix element and (ε (σ_BrF_*) − ε (π_CC_)) is the orbital energy difference. The similar equation (to Equation (1)) for the σ_H2_ → σ_BrF_* overlap may be given. NBO method was also applied here to calculate bonds’ polarizations. The natural bond orbital for the σ bond localized between atoms A and B is formed from directed orthogonal hybrids h_A_ and h_B_ [39].

σ_AB_ = c_A_ h_A_ + c_B_ h_B_, (2)

The natural hybrids are composed from a set of natural atomic orbitals. The percentage of the natural bond orbital on the A hybrid (or B hybrid may be considered), 100|c_A_|^2^ is defined as the polarization of the A-B bond; it means the percentage of the electron density on the A-atom.

The energy decomposition analysis (EDA) [40,41] was carried out with the BP86 functional [57,58], using uncontracted Slater-type orbitals (STOs) as basis functions for all of the elements, with triple-ζ quality (ADF-basis set TZP). The EDA analysis was performed for all of the complexes analyzed here and is characterized by geometries resulting from the MP2/aug-cc-pVTZ optimizations; the program package ADF2013.01 [59] was used for EDA calculations. The EDA method follows the energy partition of Morokuma [40,41], and it focuses on the instantaneous interaction energy, ΔE_int_, between two fragments (A and B) in a bond A–B, in the particular electronic reference state and in the frozen geometry of AB. This interaction energy is divided into three main components and the additional dispersion term, ΔE_disp_ (Equation (3)).

ΔE_int_ = ΔE_elstat_ + ΔE_Pauli_ + ΔE_orb_ + ΔE_disp_, (3)

The term ΔE_elstat_ corresponds to the quasiclassical electrostatic interaction between the unperturbed charge distributions of the prepared atoms and it is usually attractive. The Pauli repulsion, ΔE_Pauli_, is the energy change associated with the transformation from the superposition of the unperturbed electron densities of the isolated fragments to the wavefunction, which properly obeys the Pauli principle through explicit antisymmetrization and renormalization of the product wavefunction. This term comprises the destabilizing interactions between electrons of the same spin on either fragment. The orbital interaction, ΔE_orb_, accounts for the charge transfer and polarization effects.

## 4. Conclusions

A new kind of halogen bond was analyzed here; i.e., an interaction where the multivalent halogen plays a role of the Lewis acid centre, while the π-electron or σ-electron systems act as Lewis bases. It is interesting that those interactions possess numerous characteristics that are common with other interactions, especially hydrogen bonds as well as σ-hole and π-hole bonds. In general, these halogen bonds are steered by two main mechanisms, electrostatic interactions that play a role in arrangement of monomers in complexes, and processes that are connected with the electron charge shifts, especially from the Lewis base unit to the Lewis acid. However, it was found that the electrostatic forces are no so important in arrangement of monomers for systems that are considered if the total interactions are extremely strong [9,32]. The interactions in the complexes analyzed here are rather medium in strength, although for complexes with benzene, the –E_int_ values of ~9 kcal/mol are observed.

**Figure 5:**
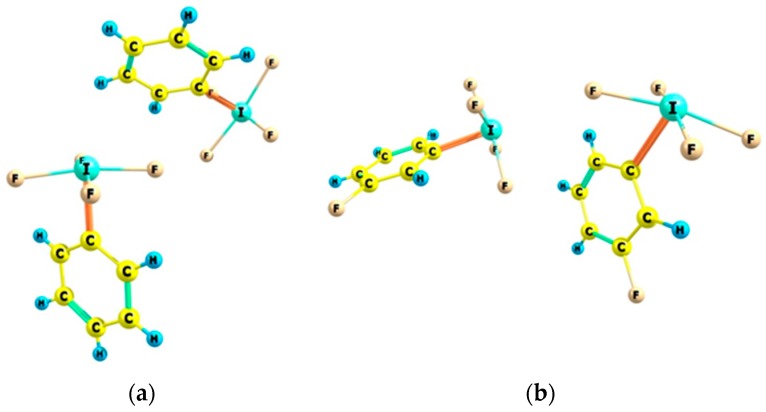
Two fragments of the crystal structures; (**a**) NACSQA refcode; (**b**) OJEDUE refcode.

It is worth mentioning that halogen bonds that are analyzed theoretically here are not so common, the preliminary search through the Cambridge Structural Database, CSD [29], was performed here (more detailed studies are in progress), and it was found such interactions are rather rare; Figure 5 presents examples of fragments of two crystal structures where the interaction of multivalent iodine with benzene π-electrons may be considered.

## Figures and Tables

**Table 1:** The energetic parameters of complexes analyzed (all in kcal/mol); interaction energy, E_int_, binding energy, E_bin_, deformation energy, E_def_, Natural Bond Orbital (NBO) energy, E_NBO_ and the BSSE correction; the distance between Lewis base and Lewis acid units is included (in Å).

Complex	Distance	E_int_	E_bin_	E_def_	BSSE	E_NBO_
BrF_3_–H_2_	2.969	−0.9	−0.8	0.0	0.5	0.6
BrF_3_–C_2_H_2_	2.904	−5.9	−5.6	0.3	1.3	6.0
BrF_3_–C_2_H_4_	2.848	−6.4	−5.9	0.5	1.8	8.6
BrF_3_–C_6_H_6_	2.845	−8.8	−8.3	0.4	3.2	10.5
BrF_5_–H_2_	3.197	−0.6	−0.6	0.0	0.4	0.2
BrF_5_–C_2_H_2_	3.218	−3.7	−3.6	0.1	1.1	1.2
BrF_5_–C_2_H_4_	3.204	−3.9	−3.8	0.1	1.5	1.6
BrF_5_–C_6_H_6_	2.911	−9.1	−8.7	0.4	3.5	2.8

**Table 2:** The interaction energy decomposition terms (in kcal/mol); Pauli repulsion, ΔE_Pauli_, electrostatic, ΔE_elstat_, orbital, ΔE_orb_, dispersion, ΔE_disp_, the total interaction energy, ΔE_int_ (in kcal/mol) and the ratio between electrostatic and orbital terms.

Complex	ΔE_Pauli_	ΔE_elstat_	ΔE_orb_	ΔE_disp_	ΔE_int_	ΔE_elstat_/ΔE_orb_
BrF_3_–H_2_	2.6	−1.4	−1.3	−1.0	−1.1	1.0
BrF_3_–C_2_H_2_	16.4	−10.6	−10.3	−2.1	−6.5	1.0
BrF_3_–C_2_H_4_	21.3	−12.4	−13.6	−2.9	−7.6	0.9
BrF_3_–C_6_H_6_	23.0	−12.1	−13.3	−5.7	−8.1	0.9
BrF_5_–H_2_	1.9	−0.9	−0.7	−1.0	−0.7	1.3
BrF_5_–C_2_H_2_	5.9	−5.4	−7.1	−2.5	−9.1	0.8
BrF_5_–C_2_H_4_	9.3	−6.2	−4.7	−3.5	−5.0	1.3
BrF_5_–C_6_H_6_	18.9	−10.8	−8.4	−7.8	−8.2	1.3

**Table 3:** The Quantum Theory of ‘Atoms in Molecules’ (QTAIM) parameters (in au) of bond critical point (BCP) of the Lewis acid—Lewis base bond path; electron density at BCP, *ρ*_BCP_, its laplacian, ∇^2^*ρ*_BCP_, the total electron energy density at BCP, H_BCP_, kinetic, and potential energy components of the latter value, G_BCP_, and V_BCP_, respectively.

Complex	*ρ*_BCP_	∇^2^*ρ*_BCP_	G_BCP_	V_BCP_	H_BCP_
BrF_3_–H_2_	0.008	0.027	0.006	−0.005	0.001
BrF_3_–C_2_H_2_	0.021	0.055	0.013	−0.013	0.000
BrF_3_–C_2_H_4_	0.024	0.053	0.014	−0.014	−0.001
BrF_3_–C_6_H_6_	0.022	0.056	0.014	−0.014	0.000
BrF_5_–H_2_	0.005	0.019	0.004	−0.003	0.001
BrF_5_–C_2_H_2_	0.013	0.036	0.008	−0.007	0.001
BrF_5_–C_2_H_4_	0.013	0.034	0.008	−0.007	0.001
BrF_5_–C_6_H_6_	0.013	0.042	0.009	−0.008	0.001

**Table 4:** The electron charge parameters; TR^NBO^ (in au) is the NBO electron charge transfer from the Lewis base to the Lewis acid; TR^H^ is the same transfer (au) but calculated from Hirshfeld charges; Q_Br_ is the NBO charge of bromine (au), POL% is the mean percentage increase of the Br-F equatorial bond polarization.

Complex	TR ^NBO^	Q_Br_^1^	TR^H^	POL%
BrF_3_–H_2_	−0.002	1.505	−0.009	1.5
BrF_3_–C_2_H_2_	−0.059	1.500	−0.088	8.5
BrF_3_–C_2_H_4_	−0.089	1.479	−0.113	9.2
BrF_3_–C_6_H_6_	−0.070	1.494	−0.114	-
BrF_5_–H_2_	−0.004	2.445	−0.003	0.5
BrF_5_–C_2_H_2_	−0.012	2.461	−0.040	3.3
BrF_5_–C_2_H_4_	−0.020	2.457	−0.049	3.5
BrF_5_–C_6_H_6_	−0.019	2.465	−0.064	4.1

^1^ Br charges in isolated BrF_3_ and BrF_5_ moieties are equal to 1.498 au and 2.439 au, respectively.

## Supplementary Materials

Supplementary materials are available online.
