# Supplementary material for: New Type of Halogen Bond: Multivalent Halogen Interacting with π- and σ-Electrons
Source: Molecules. 2017 Dec 5;22(12):2150. doi: 10.3390/molecules22122150 (PMC6149982; doi:10.3390/molecules22122150)
Supplement: Supplementary file 1 [file molecules-22-02150-s001.pdf]

molecules

Supporting Material

New Type of Halogen Bond: Multivalent Halogen Interacting with π- and σ-Electrons

Sławomir J. Grabowski [1,2]

[1] Faculty of Chemistry, University of the Basque Country and Donostia International Physics Center (DIPC), P.K. 1072, 20080 Donostia (Spain); s.grabowski@ikerbasque.org; Tel.: +34-943-01-5477
[2] IKERBASQUE, Basque Foundation for Science, 48011 Bilbao (Spain)

Abstract: MP2/aug-cc-pVTZ calculations were performed for complexes of BrF_3 and BrF_5 acting as Lewis acids through the bromine centre with species playing a role of Lewis base: dihydrogen, acetylene, ethylene and benzene. The molecular hydrogen donates electrons by its σ-bond while in remaining moieties - in complexes of hydrocarbons, such an electron transfer follows from π-electrons. The complexes are linked by a kind of the halogen bond that is analyzed first time in this study, i.e. it is the link between the multivalent halogen and π or σ-electrons. The nature of such halogen bond is discussed as well as various dependencies and correlations are presented. Different approaches are applied here, the Quantum Theory of Atoms in Molecules, Natural Bond Orbital method, the decomposition of the energy of interaction, the analysis of electrostatic potentials, etc.

Keywords: electron charge shifts; halogen bond; octet rule; hydrogen bond; σ-hole bond

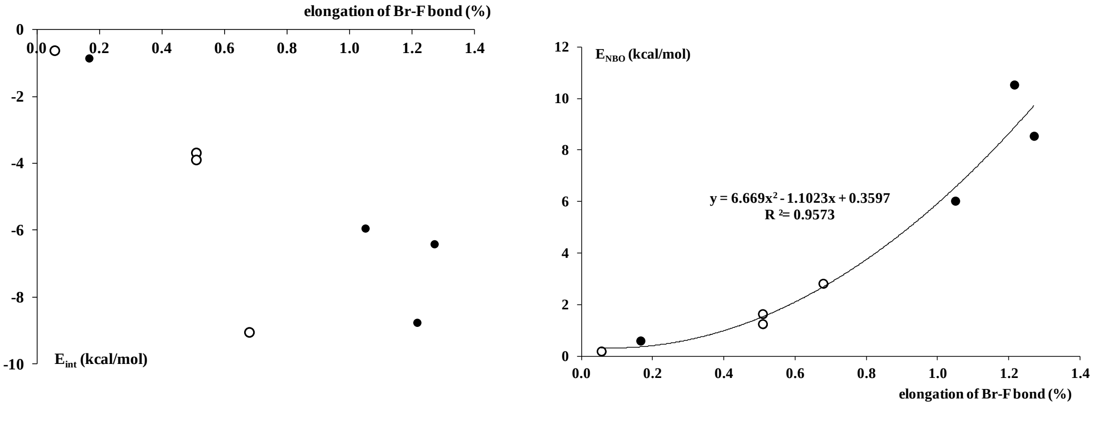

(a) (b)

Figure S1. The relationships between the elongation of the equatorial Br-F bond (percentage elongation in relation to this bond in the isolated BrF_3 or BrF_5 species) and (a) the interaction energy (corrected for BSSE); (b) the NBO energy expressing orbital-orbital interactions. Black circles correspond to the BrF_3 complexes while white ones to BrF_5 complexes.

Table S1. The equatorial (Eq) and axial (Ax) Br-F bond lengths for the BrF_3 and BrF_5 species; these bond lengths in complexes and in isolated moieties are presented in the table (all values in Å).

Complex	Eq(complex)	Ax(complex)	Eq(monomer)	Ax(monomer)
BrF_3-H_2	1.813	1.720	1.810	1.720
BrF_3-C_2H_2	1.829	1.730	1.810	1.720
BrF_3-C_2H_4	1.833	1.738	1.810	1.720
BrF_3-C_6H_6	1.832	1.737	1.810	1.720
BrF_5-H_2	1.767	1.702	1.766	1.702
BrF_5-C_2H_2	1.775	1.701	1.766	1.702
BrF_5-C_2H_4	1.775	1.702	1.766	1.702
BrF_5-C_6H_6	1.778	1.708	1.766	1.702

Table S2. The coordinates (in Å) of atoms′ positions for optimized complexes that are analyzed here are presented below; MP2/aug-cc-pVTZ level.

BrF_3-H_2

Center	Atomic	Coordinates (Angstroms)		
Number	Number	X	Y	Z
1	35	0.000000	0.193347	0.000000
2	9	-1.773223	-0.188527	0.000000
3	9	1.808872	0.301879	0.000000
4	9	0.232020	-1.511295	0.000000
5	1	-1.509261	2.697637	0.000000
6	1	-0.899767	3.116691	0.000000

BrF_3-C_2H_2

Center	Atomic	Coordinates (Angstroms)		
Number	Number	X	Y	Z
1	35	0.000000	-0.304547	0.000000
2	9	1.826015	-0.412208	0.000000
3	9	-1.826005	-0.411821	0.000000
4	9	-0.000183	-2.034275	0.000000
5	6	0.607783	2.599957	0.000000
6	6	-0.607559	2.599851	0.000000
7	1	1.671884	2.592514	0.000000
8	1	-1.671662	2.592521	0.000000

BrF_3-C_2H_4

Center	Atomic	Coordinates (Angstroms)		
Number	Number	X	Y	Z
1	35	0.359279	0.000539	0.000107
2	9	0.479496	1.829685	0.000150

3	9	0.450945	-1.830285	0.000156
4	9	2.096862	-0.013335	-0.000447
5	6	-2.491388	-0.663599	-0.000187
6	1	-2.484169	1.239496	-0.923907
7	6	-2.486238	0.677048	-0.000177
8	1	-2.484407	1.239119	0.923772
9	1	-2.493171	-1.226203	0.923341
10	1	-2.492973	-1.226547	-0.923518

BrF_3-C_6H_6

Center	Atomic	Coordinates (Angstroms)		
Number	Number	X	Y	Z
1	35	1.025207	-0.000009	0.107003
2	9	1.174003	-1.824613	0.050677
3	9	1.174096	1.824594	0.050689
4	9	2.578867	-0.000039	-0.670701
5	6	-1.600715	0.698343	1.201413
6	6	-1.909352	1.397741	0.032194
7	6	-1.601141	-0.700867	1.199997
8	6	-2.223969	0.699327	-1.131343
9	1	-1.887238	2.478837	0.029491
10	6	-1.910188	-1.397693	0.029366
11	1	-1.357032	-1.243863	2.104128
12	6	-2.224389	-0.696731	-1.132753
13	1	-2.458159	1.240784	-2.038300
14	1	-1.888741	-2.478796	0.024473
15	1	-2.458911	-1.236204	-2.040807
16	1	-1.356347	1.239351	2.106669

BrF_5-H_2

Center	Atomic	Coordinates (Angstroms)		
Number	Number	X	Y	Z
1	35	-0.032177	-0.001490	0.201292
2	9	1.362878	-1.037295	0.522354
3	9	-1.301150	1.036365	-0.460554
4	9	0.564964	0.011494	-1.392368
5	9	0.998164	1.419816	0.404460
6	9	-0.935892	-1.418376	-0.342798
7	1	-2.396370	-0.185030	2.490677
8	1	-2.678097	0.129149	1.884257

BrF_5-C_2H_2

Center	Atomic	Coordinates (Angstroms)

Number	Number	X	Y	Z
1	35	0.255077	0.035434	-0.021914
2	9	0.812467	1.720202	0.007678
3	9	0.040901	-1.728423	0.002295
4	9	1.897993	-0.334332	0.221178
5	9	0.679023	-0.058734	-1.741660
6	9	0.184433	0.045889	1.748166
7	6	-2.987288	0.744106	-0.149521
8	6	-2.934288	-0.464256	-0.046900
9	1	-3.047867	1.801914	-0.237823
10	1	-2.883706	-1.522631	0.044440

BrF_5-C_2H_4

Center	Atomic	Coordinates (Angstroms)		
Number	Number	X	Y	Z
1	35	0.323966	0.000209	-0.064463
2	9	0.489938	1.766203	0.005428
3	9	0.481847	-1.766226	0.012294
4	9	-0.178264	0.004600	1.639056
5	9	1.165618	-0.004849	-1.627357
6	9	1.897248	-0.002134	0.584489
7	6	-2.877159	-0.667363	-0.203654
8	1	-3.154169	1.230086	-1.087905
9	6	-2.876756	0.669154	-0.206044
10	1	-2.606515	1.231115	0.678127
11	1	-2.607214	-1.226354	0.682485
12	1	-3.154896	-1.231270	-1.083510

BrF_5-C_2H_6

Center	Atomic	Coordinates (Angstroms)		
Number	Number	X	Y	Z
1	35	-0.815661	0.000028	-0.000036
2	9	-1.017798	-1.249033	-1.249700
3	9	-1.017822	1.249099	1.249618
4	9	-2.523170	0.000131	-0.000158
5	9	-1.017998	-1.250061	1.248570
6	9	-1.017628	1.250145	-1.248674
7	6	2.095189	1.207823	0.699540
8	6	2.093923	-0.001941	1.395551
9	6	2.095468	1.209680	-0.696112
10	6	2.094801	-1.209851	0.696322
11	1	2.079901	-0.003381	2.477428
12	6	2.094492	0.001776	-1.395346

13	1	2.081419	2.147057	-1.236016
14	6	2.095092	-1.207988	-0.699331
15	1	2.080200	-2.147222	1.236218
16	1	2.080935	0.003221	-2.477228
17	1	2.080724	-2.143917	-1.241731
18	1	2.080906	2.143756	1.241933
